# Mutation in the knockdown resistance gene and population genetic structure in *Culex tritaeniorhynchus* from Guizhou Province

**DOI:** 10.1186/s13071-025-07071-9

**Published:** 2025-11-21

**Authors:** Weiyi Li, Zhihao Liu, Xiaomin Tang, Chengyao Liu, Lingnan Wang, Kai Deng, Libo Liu, Jiahong Wu

**Affiliations:** 1 School of Public Health, Key Laboratory of Environmental Pollution Monitoring and Disease Control, Ministry of Education, Guizhou Medical University, Guiyang, 561113 China; 2 Key Laboratory of Modern Pathogen Biology and Characteristics, Basic Medical College, Guizhou Medical University, Guiyang, 561113 Guizhou China; 3 Department of Human Parasitology, Basic Medical College, Guizhou Medical University, Guiyang, 561113 Guizhou China; 4 Guizhou Provincial Center for Disease Control and Prevention, Guiyang, 550001 Guizhou China

**Keywords:** *Culex tritaeniorhynchus*, *kdr* gene, *mtDNA–COI* gene, Population genetics, Japanese encephalitis vector, Pyrethroid resistance

## Abstract

**Background:**

*Culex tritaeniorhynchus* is the main vector of Japanese encephalitis virus. However, there is a gap in current research on the knockdown resistance gene (*kdr*) and population genetic structure of *Culex tritaeniorhynchus* in Guizhou Province, China.

**Methods:**

We collected 365 *Culex tritaeniorhynchus* mosquitoes from ten geographic populations in Guizhou Province in 2023–2024 and analyzed the genetic diversity of the *kdr* gene with mutation at locus 1014 and the mitochondrial DNA–cytochrome c oxidase subunit I (*mtDNA–COI*) gene by polymerase chain reaction (PCR) amplification and sequencing. Haplotype diversity and nucleotide diversity were also analyzed, and a haplotype network diagram and phylogenetic tree were constructed.

**Results:**

Only the L1014F mutation (TTA → TTT) was detected at locus 1014 of the *kdr* gene in *Culex tritaeniorhynchus* in Guizhou Province. The frequency of resistant alleles ranged from 0% to 8.8%, with higher frequencies observed in Dejiang (8.8%), Libo (7.1%), and Sandu (3.3%). All samples from the Xingren population were susceptible (SS). mtDNA–COI genes showed high haplotype diversity (Hd = 0.989), low nucleotide diversity (*π* = 0.023), low genetic differentiation among populations (Fst = 0.001–0.140), and high gene flow (Nm > 1), suggesting that the population of *Culex tritaeniorhynchus* in Guizhou Province has high genetic diversity and frequent gene exchange. The haplotype network and phylogenetic tree indicated that possible cryptic or novel species existed in Guizhou Province in addition to the dominant populations.

**Conclusions:**

Overall, *Culex tritaeniorhynchus* has not yet developed widespread resistance to pyrethroid insecticides in Guizhou Province, and the risk of spreading resistance genes needs to be continuously monitored owing to high population genetic diversity and frequent gene exchange.

**Graphical Abstract:**

**Figure:**
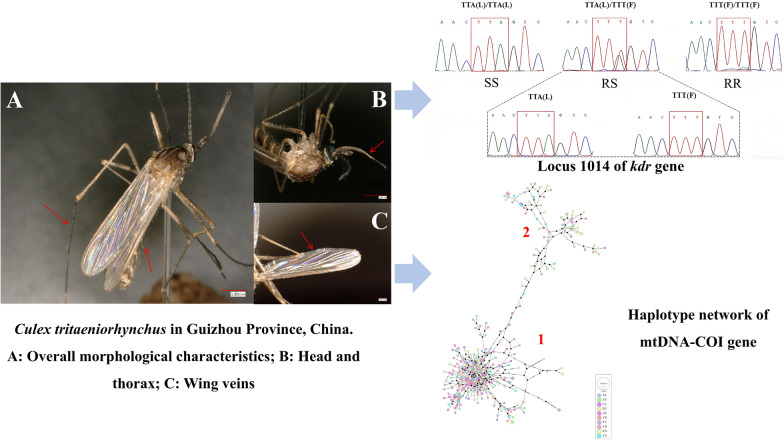

**Supplementary Information:**

The online version contains supplementary material available at 10.1186/s13071-025-07071-9.

## Background

*Culex tritaeniorhynchus* is a mosquito vector that is widely distributed in tropical and subtropical regions of Asia, including China, Japan, India, and Southeast Asia [1]. The eastern, central, and southwestern regions of China are highly suitable habitats for *Culex tritaeniorhynchus* [2]. Guizhou Province, with its humid climate and dense rice paddies, provides an ideal breeding ground for *Culex tritaeniorhynchus*. Data from the Chinese Center for Disease Control and Prevention (CDC) show that the incidence of Japanese encephalitis virus (JEV) in Guizhou Province has long been higher than the national average. *Culex tritaeniorhynchus* is not only the main vector of JEV, but also transmits a variety of other viral diseases, such as West Nile fever, which is a serious threat to human health and public health safety [3–5].

The use of chemical insecticides has long been the mainstay of mosquito control [6]. Currently, the main insecticides commonly used to control mosquitoes and other important pests include organophosphates, carbamates, and pyrethroids [7]. Although the application of a large number of chemical insecticides has effectively reduced the population density of *Culex tritaeniorhynchus*, the long-term use of these insecticides has also led to an increase in the resistance of *Culex tritaeniorhynchus*, and the number of related reports continues to increase. For example, mutations in the voltage-gated sodium channel of knockdown resistance (*kdr*) and mutations in the gene region of ace-1 acetylcholinesterase (*ace-1*) are well known as resistance target mutations [8]. One study detected the *Ace-1* F331W mutation, which was significantly associated with organophosphorus resistance, in *Culex tritaeniorhynchus* populations collected from different regions of China [9]. An in-depth study of 12 populations of *Culex tritaeniorhynchus* obtained from China was also conducted and found that the frequency of *kdr* mutation fluctuated between 10% and 29.6% and that there was a significant positive correlation between resistance to pyrethroid insecticides and the frequency of *kdr* allele [10]. *Culex tritaeniorhynchus* has also been found to exhibit high levels of resistance to insecticides in other countries [11, 12]. In Guizhou Province, China, we also investigated the resistance of *Anopheles sinensis* and found high levels of resistance in different areas. (unpublished data). *Anopheles sinensis* shares the same habitat as *Culex tritaeniorhynchus*, suggesting that resistance may be widespread among *Culex tritaeniorhynchus* in Guizhou Province. However, there are few reports on the systematic study of insecticide resistance of *Culex tritaeniorhynchus* in Guizhou Province.

Population genetics focuses on the study of the drivers of gene frequency changes, and in the field of vector mosquito control, the genetic structure of populations directly affects the efficacy of disease transmission and the development of resistance in mosquito vectors [13]. Studies have shown that the level of gene flow is significantly and positively correlated with the spread of resistance genes: high gene flow promotes the cross-population spread of resistance genes, while genetic isolation can facilitate the emergence of localized resistance adaptations [13, 14].

In this study, we collected *Culex tritaeniorhynchus* from different regions of Guizhou Province. Molecular biology methods were used to detect variations in the *kdr* gene locus and their geographical distribution. At the same time, mitochondrial DNA–cytochrome c oxidase subunit I (*mtDNA–COI*) gene analysis was used to analyze the genetic structure of the population. Finally, the two were combined to explore genetic diversity and gene flow patterns. The results provide basic data for drug resistance monitoring and population genetics of *Culex tritaeniorhynchus* in Guizhou Province, as well as a reference for local mosquito-borne disease prevention and control strategies.

## Methods

### Sample collection and identification

Mosquitoes were collected from July to October 2023–2024 at ten sampling sites in Guizhou Province using mosquito suction devices and mosquito trapping lamps (Table 1 and Supplementary Fig. S1). After collection, the mosquitoes were identified on the basis of standard morphological features, and *Culex tritaeniorhynchus* was initially screened. According to the collection location and mosquito species, the samples were stored in 2-mL freezing tubes and brought back to the laboratory and immediately flash-frozen in liquid nitrogen. Detailed records of specimen name, number, collection location, place, time, and collector were recorded to ensure the completeness and traceability of sample information. Morphologically identified specimens were further confirmed via mtDNA–COI sequencing.

**Table 1:** Information on the collection sites of wild populations of *Culex tritaeniorhynchus* in Guizhou Province, China

Location	Longitude, latitude	Habitat conditions	Collection time	Code
Anlong County, Qianxinan Prefecture	25.099° N, 105.442° E	Cattle pens	August 2024	AL
Anshun City	26.402° N, 106.451° E	Pig pens, cattle pens	July 2024	AS
Congjiang County, Qiandongnan Prefecture	25.737° N, 108.907° E	Cattle pens	July 2024	CJ
Dejiang County, Tongren City	28.550° N, 108.162° E	Pig pens	July 2023	DJ
Libo County, Qiannan Prefecture	25.390° N, 108.072° E	Cattle pens	September 2023	LB
Nayong County, Bijie City	27.006° N, 105.199° E	Pig pens	July 2023	NY
Sandu County, Qiannan Prefecture	25.610° N, 108.018° E	Cattle pens	August 2024	SD
Weining County, Bijie City	26.867° N, 104.282° E	Cattle pens	August 2023	WN
Xingren City, Qianxinan Prefecture	25.683° N, 105.446° E	Pig pens	July 2024	XR
Xishui County, Zunyi City	28.223° N, 106.108° E	Woods	July 2023	XS

### DNA extraction and polymerase chain reaction amplification

Mosquito genomic DNA was extracted using TaKaRa MiniBEST Universal Genomic DNA Extraction Kit, version 5.0. *mtDNA–COI* gene-specific primers (COI-F: GGTCAACAAATCATAAAGATATTGG; COI-R: TAAACTTCAGGGGTGACCAAAAAATCA) were obtained from Folmer [15]. Primers were synthesized by Sangon Biotech (Shanghai) Co. PCR amplification was carried out in 25-μl reaction volumes consisting of 12.5 μl PCR mixture, 1.0 μl of each primer, 1.5 μl of template DNA, and 9.0 μl of DDH_2_O. The amplification program consisted of 95 °C for 5 min, followed by 35 cycles of 95 °C for 30 s, 51 °C for 30 s, and 72 °C for 1 min, and one cycle of 72 °C for 5 min. On the basis of the kdr gene fragment reported in the literature [11], we used National Center for Biotechnology Information (NCBI) primer-blast (https://www.ncbi.nlm.nih.gov/tools/primer-blast) to redesign specific primers of appropriate size for the *kdr* gene (*kdr*-F: CTTCACCGACTTCATGCACTC; *kdr*-R: GATTTTGGGACAAAAGCAAGGC) for amplifying a fragment of the *kdr* gene (325 bp), and parameters matched those used for *mtDNA–COI* amplification, except for an annealing temperature of 57 °C. The PCR products were electrophoresed on 1.0% agarose gel and sent to Sangon Biotech (Shanghai) Co. for Sanger sequencing. To confirm the mutation status of the sequence map at the 1014 site in the heterozygous sample, heterozygous samples were cloned using pClone007 Versatile Simple Vector and transferred into DH5a receptor cell culture, and a single colony clone was picked and sequenced for verification.

### Data analysis

We used Chromas software (https://chromas.updatestar.com/download) to determine whether the sequencing results were normal and to identify *kdr* gene mutations. For specific resistance-related genotypes, this was calculated by counting the number of individuals with the genotype and dividing by the overall number of individuals. Allele frequencies were calculated using standard heterozygote adjustment formulas: S% = SS% + 0.5 × RS% and R% = RR% + 0.5 × RS%, where SS is sensitive pure, RR is resistant pure, and RS is resistant heterozygous.

On the basis of the *mtDNA–COI* sequences, haplotype diversity (Hd) and nucleotide diversity (*π*) were calculated, and three statistical methods, Tajima’s *D*, Fu and Li’s *F*, and Fu and Li’s *D*, were selected for neutrality testing using DnaSP, version 6 (http://www.ub.edu/dnasp/). Genetic differentiation coefficient (Fst) and gene flow (Nm) were analyzed using Arlequin, version 3.5.2 (https://cmpg.unibe.ch/software/arlequin35/). Haplotype network diagrams were constructed using PopART, version 1.7, software (https://popart.maths.otago.ac.nz). Genetic distances between various populations were calculated, and phylogenetic trees were constructed by the maximum likelihood method using MEGA, version 11.0 (https://www.megasoftware.net) with a bootstrap value of 1000.

## Results

### Mitochondrial DNA genetic diversity and genetic differentiation

For all *Culex tritaeniorhynchus* specimens in which the *kdr* gene was detected, *mtDNA–COI* sequences (685 bp) were simultaneously obtained. The average content of *mtDNA–COI* bases in the ten populations was 39.6% A, 15.4% T, 29.3% C, and 15.7% G, which was consistent with the characterization of mitochondrial genes. A total of 267 haplotypes were detected in 365 sequences, with 12.0% shared haplotypes. The haplotype diversity (Hd) was 0.989, approaching the maximum haplotype richness, and the nucleotide diversity (Pi) was 0.023, indicating moderate nucleotide variation. Significant negative deviations from neutrality were observed only when all populations were considered collectively, and the *Culex tritaeniorhynchus* populations in Guihzou may have experienced regional expansion development (Table 2). In addition, the intrapopulation genetic distances of the ten populations ranged from 0.01 to 0.03, and the interpopulation genetic distances ranged from 0.015 to 0.034 (Supplementary Table S1); the Fst ranged from 0.001 to 0.140, and the gene flow Nm was greater than 1 in all of them—Nm > 1 generally implies sufficient gene flow to prevent drift-based differentiation (Supplementary Table S2). It indicated that different populations of *Culex tritaeniorhynchus* in Guizhou Province had lower genetic differences and higher gene exchange.

**Table 2:** Genetic diversity of different populations of *Culex tritaeniorhynchus* populations in Guizhou Province, China

Population	Sample size (*n*)	Single mutation site	Parsimony-informative site	Nucleotide diversity	Number of haplotypes	Haplotype diversity	Tajima’s *D*		Fu and Li’s *F*		Fu and Li’s *D*	
							*D*	*P*	Fs	*P*	*D*	*P*
AL	42	22	39	0.01334	36	0.985	−1.574	> 0.10	−1.574	−1.574	> 0.10	−1.574
AS	46	32	41	0.0239	43	0.996	−0.598	> 0.10	−1.927	−0.598	> 0.10	−1.927
CJ	31	25	41	0.02188	27	0.987	−0.725	> 0.10	−1.275	−0.725	> 0.10	−1.275
DJ	34	39	64	0.03305	31	0.993	−0.898	> 0.10	−1.414	−0.898	> 0.10	−1.414
LB	35	14	45	0.02075	32	0.995	−0.306	> 0.10	−0.172	−0.306	> 0.10	−0.172
NY	18	26	59	0.03121	14	0.954	−1.043	> 0.10	−0.54	−1.043	> 0.10	−0.54
SD	39	27	38	0.01363	32	0.981	−1.614	> 0.05	−1.78	−1.614	> 0.05	−1.78
WN	48	72	50	0.03031	43	0.993	−1.197	> 0.10	−3.294	−1.197	> 0.10	−3.294
XR	49	24	44	0.01653	41	0.985	−1.157	> 0.10	−1.554	−1.157	> 0.10	−1.554
XS	23	8	39	0.02414	22	0.996	0.723	> 0.10	0.579	0.723	> 0.10	0.579
Total	365	77	128	0.02323	267	0.989	−1.903	< 0.05^*^	−4.42	−1.903	< 0.05^*^	−4.42

### Haplotype network and phylogenetic analysis

**Fig. 1:**
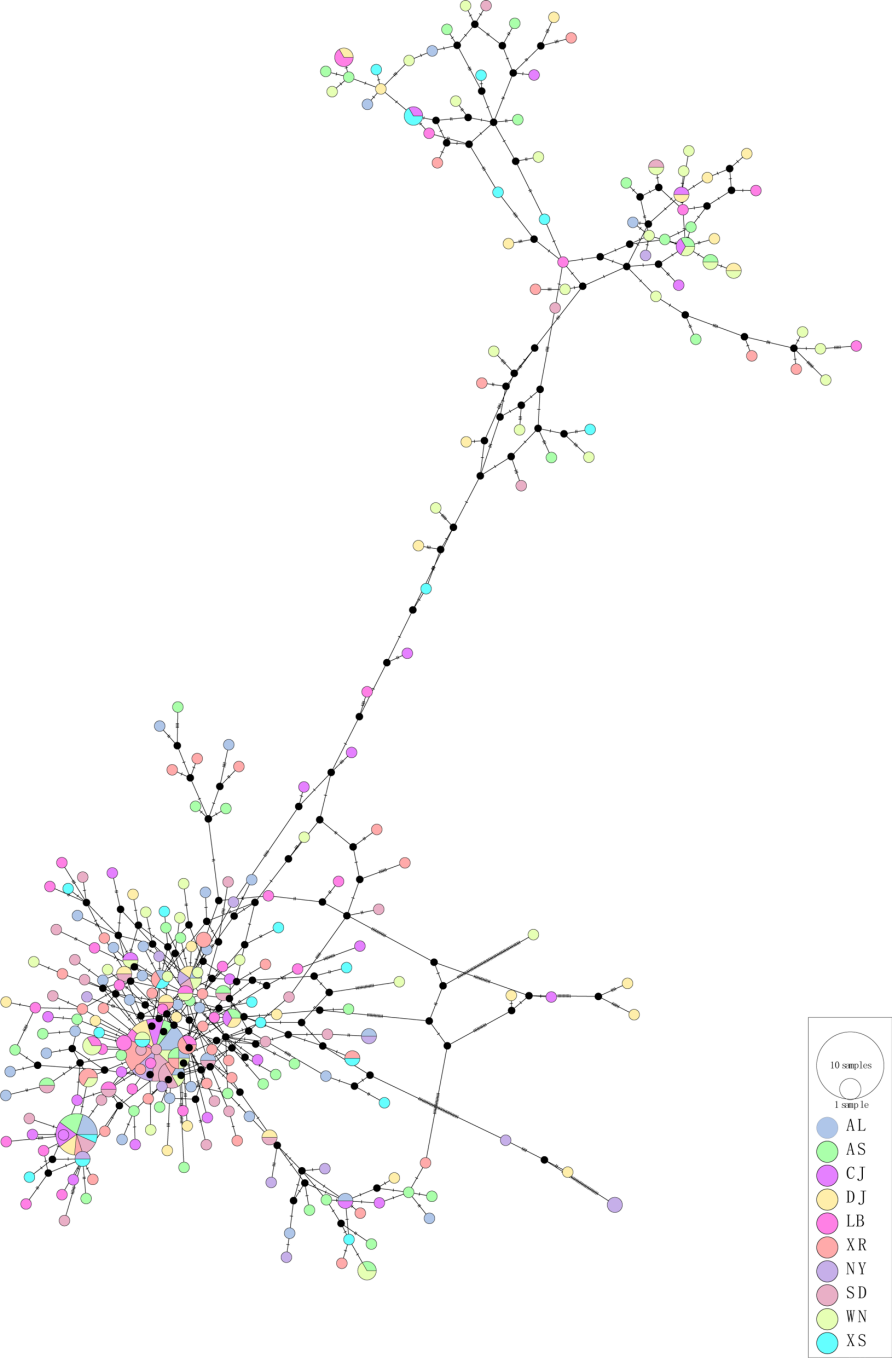
Haplotype network of *mtDNA–COI* gene in different populations of *Culex tritaeniorhynchus* in Guizhou Province

A haplotype network was constructed from the haplotype sequences (Fig. 1), showing the distribution of 267 haplotypes in the *Culex tritaeniorhynchus* population in Guizhou Province. In the *Culex tritaeniorhynchus* population in Guizhou Province, two distinct groups were identified: group 1, which was more distinct, and group 2, which was more dispersed. Haplotype Hap3 was dominant in group 1, with 34 haplotypes. This was followed by Hap6, with 15 haplotypes. These results suggest that the haplotypes are ancestral or have recently expanded. Group 2 did not have a clearly dominant haplotype. At the regional level, the haplotypes of the ten regions did not show significant clustering according to the regions, neither in group 1 nor in group 2.

**Fig. 2:**
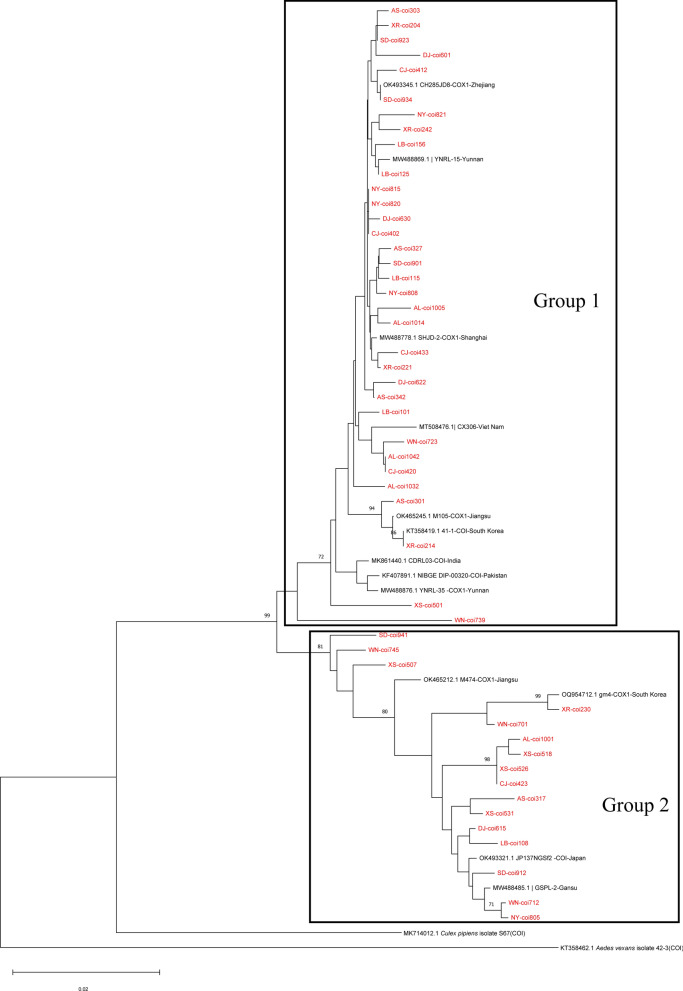
Phylogenetic tree based on the *mtDNA–COI* gene of *Culex tritaeniorhynchus*

Five random *mtDNA–COI* sequences per region were selected to participate in the construction of the phylogenetic tree (Fig. 2). All *Culex tritaeniorhynchus* samples were clustered into two major branches. *Culex tritaeniorhynchus* samples from different regions of Guizhou Province did not show obvious geographical aggregation but were evenly dispersed in the two branches. This supports the hypothesis of high population mobility or gene flow across Guizhou. In branch 1, the samples showed some aggregation from the Yunnan region of China and South Asia, while in branch 2, the samples showed some tendency of aggregation from the northern region of China, Japan, and Korea. Cross-branch geographic overlap suggests that both *Culex tritaeniorhynchus* groups are widely distributed across East Asia.

### *kdr* gene mutation characterization

**Fig. 3:**
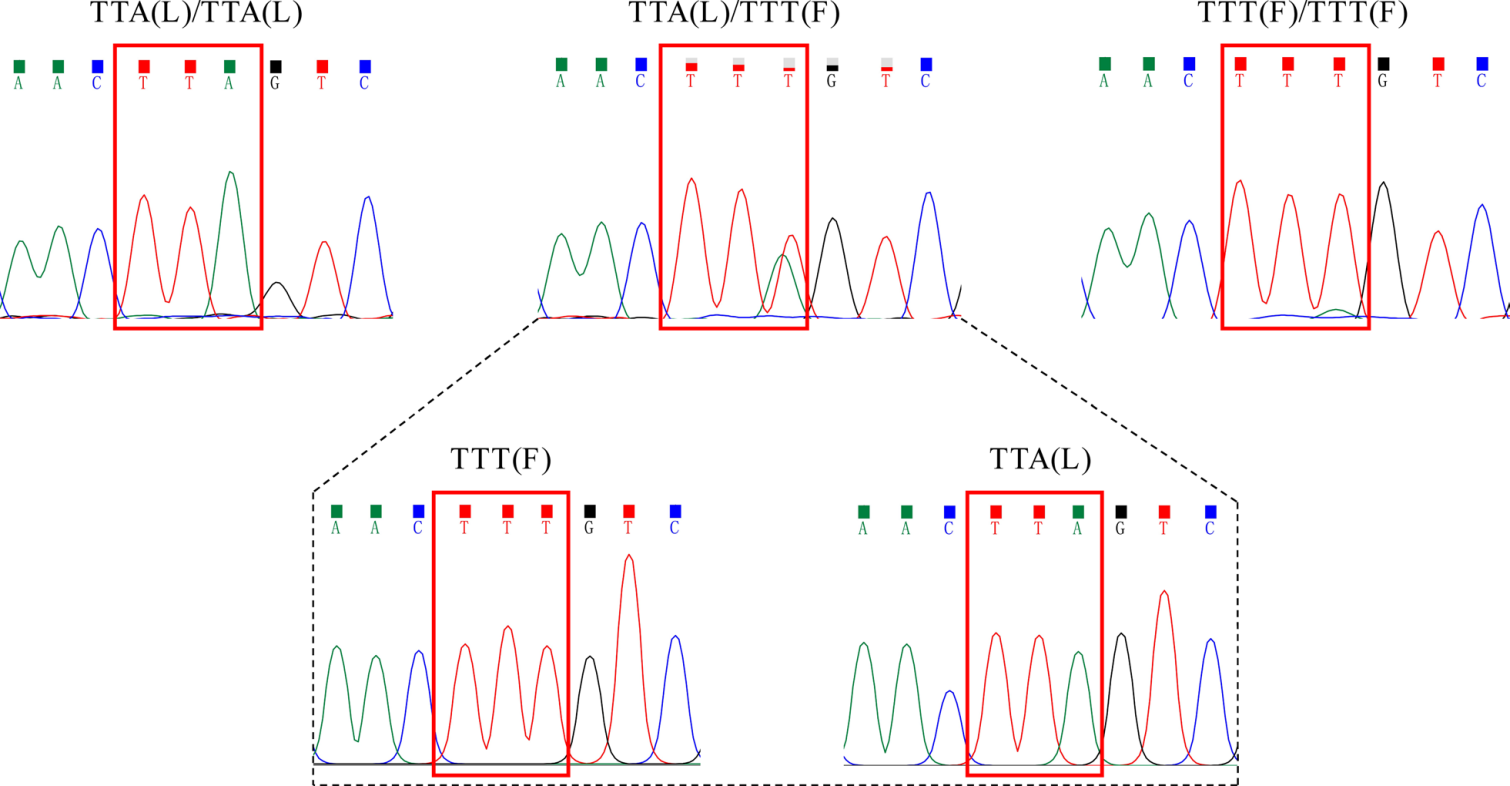
Sequencing peak map of genotype at the 1014 position of the *kdr* gene in* Culex tritaeniorhynchus*

PCR amplification of 365 *Culex tritaeniorhynchus* DNA samples yielded clear single bands. Sequencing results showed that one mutant gene, L1014F (TTA → TTT), was detected at locus 1014 (Fig. 3). Resistant heterozygotes were confirmed by clonal sequencing, and multiple colonies were confirmed to be heterozygous by the simultaneous presence of TTA and TTT in their gene sequences.

The mutation frequency of the *kdr* gene in populations across Guizhou ranged from 0% to 8.8% (Table 2), with the highest mutation frequency (8.8%) in the DJ population, and no samples exhibited resistance-associated alleles in the XR population. Resistant pure haplotypes (RR) were detected only in WN and AS populations, with 2.1 and 2.2%, respectively. Sensitive pure haplotypes (SS) accounted for more than 82% of all populations (Table 3).

**Table 3:** Statistical results of genotypes and their frequencies at locus 1014 of the *kdr* gene in *Culex tritaeniorhynchus*

Population	*n*	*kdr *(%)	Genotype					
			SS	frequency (%)	RS	frequency (%)	RR	frequency (%)
AL	42	2.38	40	95.2	2	4.8	0	0
AS	46	4.35	43	93.5	2	4.4	1	2.2
CJ	31	1.61	30	96.8	1	3.2	0	0
DJ	34	8.82	28	82.4	6	17.7	0	0
LB	35	7.14	30	85.7	5	14.3	0	0
NY	18	2.78	17	94.4	1	5.6	0	0
SD	39	3.35	36	92.3	3	7.7	0	0
WN	48	3.12	46	91.7	1	2.1	1	2.1
XR	49	0	49	100.0	0	0.0	0	0.0
XS	23	2.17	22	95.7	1	4.4	0	0.0

*S* sensitive, *R* resistant, *RS* resistant heterozygote

## Discussion

Insecticide resistance reflects adaptive evolutionary dynamics, where selective pressure favors the survival and proliferation of resistant genotypes, where sensitive individuals are gradually eliminated under the continuous action of insecticides, and resistance genes accumulate in populations through natural selection and spread to other regions with the migration of individuals [16]. Therefore, in this study, we examined the resistance genes and *mtDNA–COI* genes and analyzed the population genetic structure of *Culex tritaeniorhynchus* in Guizhou Province, to inform surveillance strategies and guide regional vector control interventions in Guizhou Province.

In this study, *mtDNA–COI* sequences (*n* = 365) were obtained for all specimens in which the *kdr* gene was successfully detected. *mtDNA–COIs* were examined and a total of 267 haplotypes were identified. Similar to some previous studies, *Culex tritaeniorhynchus* populations in Guizhou Province showed high haplotype diversity and low nucleotide diversity [17, 18]. High haplotypic diversity coupled with low nucleotide diversity suggests a young, expansive population undergoing rapid ecological adaptation. Some researchers have suggested that this environmental condition conducive to vector proliferation for mosquito survival and dispersal in tropical/subtropical regions contributes to this phenomenon [19, 20]. The high genetic diversity helps insects accumulate ecologically adapted advantageous genes and acquire mutations favoring resistance more rapidly in each generation, exacerbating the risk of insect-borne disease transmission [21].

Meanwhile, the results of genetic differentiation coefficient (Fst = 0.001–0.140) and gene flow (Nm > 1) among *Culex tritaeniorhynchus* populations in different regions of Guizhou indicated that most populations are not genetically isolated. It is also consistent with the genetic exchange of populations between other regions in mainland China [22]. This situation may be related to a variety of factors. Guizhou Province has a well-developed tourism industry, and frequent human activities and increased tourist activities may provide vectors for mosquitoes to migrate across regions [23]. The larvae and eggs of *Culex tritaeniorhynchus* may be dispersed downstream with water currents or may be transmitted over long distances by vectors such as boats [24]. Anthropogenic and hydrological factors likely contribute to the spatial dispersal and genetic homogenization of regional populations. In addition, this may also be related to the flight ability of *Culex tritaeniorhynchus* adult mosquitoes, which has been found to be able to migrate long distances relying on the monsoon and even able to transoceanic dispersal between different countries in East Asia, thus contributing to the rich genetic exchange of *Culex tritaeniorhynchus* populations between different regions and countries [25, 26]. This capacity for long-distance dispersal via prevailing winds has critical implications for cross-border resistance management. Although the mutation frequency of *Culex tritaeniorhynchus kdr* gene in Guizhou Province is currently at a low level, it needs continuous attention because of its frequent gene exchange and the ease of spreading the resistance gene to different regions after its formation.

Haplotype networks and phylogenetic trees were constructed on the basis of the *mtDNA–COI* gene, and the results of the two methods basically coincided, both indicating that the *Culex tritaeniorhynchus* population in Guizhou Province was divided into two groups. This genealogical pattern is widespread in China, Korea, and Japan, and has been suggested by some researchers to indicate the presence of possible cryptic or novel species in *Culex tritaeniorhynchus* [17, 22, 26]. These findings align with emerging evidence of cryptic speciation within *Culex tritaeniorhynchus*, though further morphometric and genomic analyses are required. In addition, at the regional level in Guizhou Province, different regions did not show significant clustering, further supporting the ability of *Culex tritaeniorhynchus* to exchange genes across regions.

The *kdr* allele gene frequency of *Culex tritaeniorhynchus* in Guizhou Province was low overall (0–8.8%), much lower than that of *Culex tritaeniorhynchus* in the neighboring region of Sichuan Province (17.0–27.8%), and also lower than that of the rest of China (10–29.6%) [10, 27]. Mosquito resistance to insecticides arises through a variety of mechanisms, including metabolic resistance, epidermal resistance, behavioral resistance, and target resistance [28]. Mutations in the target locus render the insecticide ineffective for binding or less potent, thus reducing its killing effect [29]. The *kdr* gene detected in this study is also one of the target loci, and some studies have shown that there is a positive correlation between pyrethroid resistance and *kdr* mutation frequency. This indicates that *Culex tritaeniorhynchus* populations in Guizhou Province have not yet developed widespread resistance to pyrethroid insecticides, and effective control of *Culex tritaeniorhynchus* populations can still be achieved by pyrethroid insecticides at present. However, resistance studies on other mosquitoes have also shown that metabolic resistance also plays an important role in pyrethroid insecticide resistance in *Anopheles sinensis* in some areas of China [30]. While target-site mutations such as *kdr* play a clear role, integrated resistance mechanisms—including metabolic and behavioral adaptations—are likely co-contributors. This also suggests that more data are needed to clarify the pyrethroid insecticide resistance of *Culex tritaeniorhynchus* in Guizhou Province. At the same time, on the basis of the results of *mtDNA–COI* gene, owing to the rich gene exchange among populations in different regions, resistance that appears in one region may rapidly spread to other regions. Although *kdr* resistance is currently at a low level, given the high connectivity among populations, ongoing molecular monitoring remains critical to intercept emerging resistance patterns. Local public health departments should strengthen surveillance of insecticide resistance in *Culex tritaeniorhynchus* to prevent the spread of insecticide resistance.

## Conclusions

Only one mutation type (L1014F, TTA → TTT) was identified at the 1014 locus of the *kdr* gene in *Culex tritaeniorhynchus* across Guizhou Province. Its low mutation frequency indicates that pyrethroid insecticides remain an effective control measure for local populations. However, high levels of gene flow among regions could promote rapid spread of resistance alleles once established. These findings highlight the urgency of sustained molecular surveillance and adaptive insecticide management strategies to mitigate future resistance development.

## Supplementary Information

Supplementary material 1. Additional file 1: Fig. S1 Geographical Distribution Map of Sampling Points of *Culex tritaeniorhynchus* in Guizhou Province, China. Tab.S1 Genetic distance of *Culex tritaeniorhynchus* populations in different regions based on mtDNA–COI

## Data Availability

The datasets used and analyzed during the current study are available from the corresponding author on reasonable request. FASTA files of genomes were deposited in NCBI GenBank and are available under the accession nos. PV890101—PV890465.

## Abbreviations

*kdr*: Knockdown resistance gene
*mtDNA–COI*: Mitochondrial DNA-cytochrome c oxidase subunit I gene
JEV: Japanese encephalitis virus
