# Supplementary material for: Mutation in the knockdown resistance gene and population genetic structure in Culex tritaeniorhynchus from Guizhou Province
Source: Parasit Vectors. 2025 Nov 21;18:481. doi: 10.1186/s13071-025-07071-9 (PMC12639896; doi:10.1186/s13071-025-07071-9)
Supplement: Supplementary file 1 — Supplementary material 1. Additional file 1: Fig. S1 Geographical Distribution Map of Sampling Points of Culex tritaeniorhynchus in Guizhou Province, China. Tab.S1 Genetic distance of Culex tritaeniorhynchus populations in different regions based on mtDNA–COI [file 13071_2025_7071_MOESM1_ESM.docx]

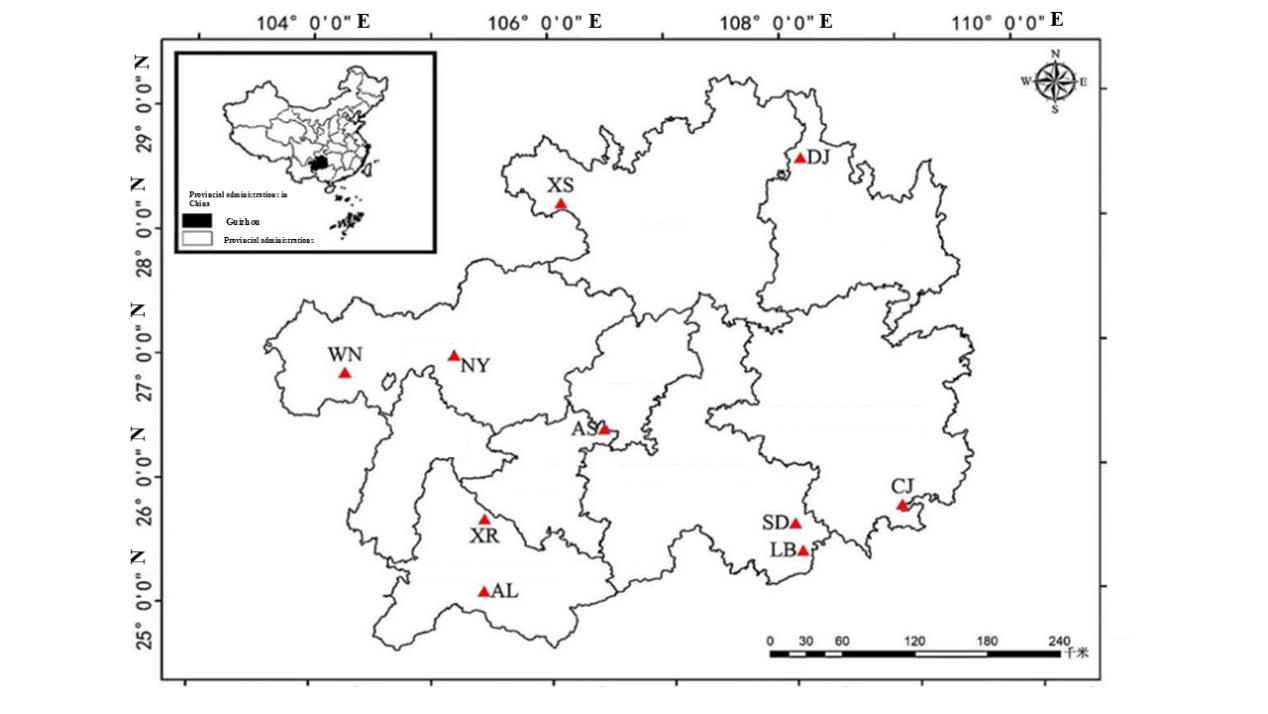


**S.Fig. 1 Geographical Distribution Map of Sampling Points of *Culex tritaeniorhynchus* in Guizhou Province, China**

**S.Tab.1 Genetic distance of Culex tritaeniorhynchus populations in different regions based on mtDNA-COI**

| Population | AL | AS | CJ | DJ | LB | NY | SD | WN | XR | XS |
| --- | --- | --- | --- | --- | --- | --- | --- | --- | --- | --- |
| AL | 0.010 |  |  |  |  |  |  |  |  |  |
| AS | 0.019 | 0.020 |  |  |  |  |  |  |  |  |
| CJ | 0.018 | 0.023 | 0.020 |  |  |  |  |  |  |  |
| DJ | 0.025 | 0.028 | 0.028 | 0.030 |  |  |  |  |  |  |
| LB | 0.017 | 0.022 | 0.021 | 0.027 | 0.020 |  |  |  |  |  |
| NY | 0.015 | 0.021 | 0.019 | 0.026 | 0.019 | 0.020 |  |  |  |  |
| SD | 0.023 | 0.029 | 0.027 | 0.034 | 0.027 | 0.025 | 0.030 |  |  |  |
| WN | 0.013 | 0.020 | 0.018 | 0.026 | 0.017 | 0.015 | 0.023 | 0.010 |  |  |
| XR | 0.025 | 0.028 | 0.027 | 0.032 | 0.027 | 0.026 | 0.034 | 0.025 | 0.030 |  |
| XS | 0.021 | 0.024 | 0.023 | 0.029 | 0.023 | 0.022 | 0.030 | 0.021 | 0.027 | 0.020 |

**S.Tab.2 Genetic differentiation index (Fst) and gene flow (Nm) among different populations**

| Population | AL | AS | CJ | DJ | LB | NY | SD | WN | XR | XS |  |
| --- | --- | --- | --- | --- | --- | --- | --- | --- | --- | --- | --- |
| AL | . | inf | 2.196 | 2.998 | 3.438 | 21.854 | inf | 33.950 | inf | 2.097 |  |
| AS | 0.000 | . | 1.637 | 2.219 | 2.093 | 5.458 | inf | 5.200 | 11.460 | 1.540 |  |
| CJ | 0.102 | 0.133 | . | 4.046 | inf | 17.736 | 2.557 | 15.921 | 4.843 | inf |  |
| DJ | 0.077 | 0.101 | 0.058 | . | 4.815 | 5.388 | 4.986 | 7.480 | 4.599 | 4.190 |  |
| LB | 0.068 | 0.107 | 0.000 | 0.049 | . | 409.586 | 3.417 | 141.795 | 8.416 | inf |  |
| NY | 0.011 | 0.044 | 0.014 | 0.044 | 0.001 | . | 14.856 | inf | inf | 11.775 |  |
| SD | 0.000 | 0.000 | 0.089 | 0.048 | 0.068 | 0.017 | . | 23.514 | 47.735 | 2.418 |  |
| WN | 0.007 | 0.046 | 0.015 | 0.032 | 0.002 | 0.000 | 0.011 | . | inf | 12.029 |  |
| XR | 0.000 | 0.021 | 0.049 | 0.052 | 0.029 | 0.000 | 0.005 | 0.000 | . | 4.922 |  |
| XS | 0.107 | 0.140 | 0.000 | 0.056 | 0.000 | 0.021 | 0.094 | 0.020 | 0.048 | . |  |

Note: The lower left corner indicates the Fst value and the upper right corner indicates the Nm value.
